# Exploring the Associations Between Affective and Non‐Affective Cognitive Domains in Mood Disorders and Healthy Controls Using Network Analysis

**DOI:** 10.1111/acps.70084

**Published:** 2026-03-03

**Authors:** Hanne Lie Kjærstad, Astrid Endrup Iversen, Maj Vinberg, Lars Vedel Kessing, Jeff Zarp, Kamilla Woznica Miskowiak

**Affiliations:** ^1^ Neurocognition and Emotion in Affective Disorders (NEAD) Centre, Mental Health Services, Capital Region of Denmark, and Department of Psychology University of Copenhagen Copenhagen Denmark; ^2^ Copenhagen Affective Disorder Research Centre (CADIC) Mental Health Center Copenhagen, Rigshospitalet Copenhagen Denmark; ^3^ Department of Clinical Medicine University of Copenhagen Copenhagen Denmark; ^4^ Early Multimodular Prevention and Intervention Research Institution (EMPIRI), Mental Health Centre, Northern Zealand, Mental Health Services, Capital Region of Denmark Hilleroed Denmark

**Keywords:** affective cognition, bipolar disorder, cognition, emotion regulation, major depressive disorder, mood disorders, network analysis

## Abstract

**Introduction:**

Cognitive impairment is a core feature of mood disorders that contributes to reduced functioning and poorer prognosis, thereby emerging as an important treatment target. Persistent trait‐related impairments present within both affective and non‐affective cognition. Nevertheless, the relationship between affective and non‐affective cognitive domains remains unclear, including whether impairments in emotion regulation and facial expression recognition are secondary to deficits in non‐affective cognition. Mapping out the hierarchical structure of affective and non‐affective cognitive domains may elucidate core cognitive impairments that represent the most relevant treatment targets.

**Methods:**

Network analysis was employed to explore the associations between affective and non‐affective cognitive domains in individuals with mood disorders (*N* = 380) and healthy controls (HC; *N* = 225) pooled from two previous studies. Partial correlation networks were constructed separately for individuals with mood disorders and HC comprising measures of non‐affective cognition (working memory and executive function, attention and processing speed, verbal learning, and verbal memory) and affective cognition (emotion regulation success, facial expression recognition speed and accuracy).

**Results:**

For both mood disorders and HC, ‘working memory and executive function’ and ‘attention and processing speed’ emerged as central cognitive domains. Emotion regulation showed a significantly weaker association with ‘working memory and executive function’ in mood disorders relative to HC. Additionally, facial expression recognition speed was associated with ‘attention and processing speed’ across both groups.

**Conclusion:**

Our findings suggest that working memory, executive function, attention, and processing speed are core cognitive domains in mood disorders. Further, the weak association between executive function and emotion regulation in mood disorders may indicate a reduced reliance on cognitive control processes during emotion regulation. These findings underscore the importance of targeting both affective and non‐affective cognition in pro‐cognitive interventions to improve emotion regulation and potentially mitigate the risk of mood episodes.

## Key Points

- Working memory, executive function, attention, and processing speed emerged as central cognitive domains in mood disorders and healthy controls.
- Emotion regulation was weakly linked with working memory and executive function in mood disorders, suggesting that emotion regulation strategies may rely less on cognitive control mechanisms in individuals with mood disorders.
- Facial expression recognition speed was associated with attention and processing speed across mood disorders and healthy controls.

**Limitations:**

- Cognitive test scores were grouped into composite measures to accommodate for differences in the neurocognitive tests used in the included studies.
- Limited measures of affective cognition.
- Most individuals with mood disorders were prescribed psychotropic medication, which could influence cognition.
- The cross‐sectional design of the study hampers causal inferences regarding the directionality of influence between domains.

## 1 Introduction

Mood disorders, including major depressive disorder (MDD) and bipolar disorder (BD), are often disabling conditions characterized by mood fluctuations and multiple relapses [1, 2]. Cognitive impairments are increasingly recognized as a core feature of these disorders, affecting approximately half of affected individuals [3, 4]. These deficits not only persist beyond mood episodes but are also present in the early stages of the disorders and unaffected relatives, suggesting a potential trait marker with familial risk [5, 6, 7, 8]. Moreover, cognitive impairments contribute to reduced functioning and poorer treatment response and prognosis, including a higher risk of illness onset and future relapse and hospitalizations in those at risk and affected individuals, respectively [9, 10, 11, 12]. Taken together, this highlights the urgent need for early prophylactic strategies and interventions targeting cognition to mitigate the risk of illness onset and adverse illness course.

Individuals with mood disorders exhibit persistent trait‐related impairments within both *non‐affective cognition*—including attention, processing speed, memory, and executive function [13, 14]—as well as in *affective cognition*—including emotion processing and regulation and facial expression recognition [15, 16]. Despite traditionally being studied separately, growing evidence suggests important ties between non‐affective and affective cognition in mood disorders [17]. Indeed, subgroups of individuals with BD with non‐affective cognitive impairments present with greater difficulties with facial expression recognition and emotion regulation relative to those with intact non‐affective cognition [18, 19]. Across mood disorders, impairments in emotion recognition and regulation appear to be at least partially mediated by attention‐executive deficits [20, 21]. These findings are supported by neuroimaging research showing substantial overlap in brain regions involved in affective and non‐affective cognitive processes, particularly involving the dorsal and medial prefrontal regions and limbic structures [22, 23]. This suggests that disruptions in these shared networks may underlie the co‐occurring impairment in affective and non‐affective cognition in mood disorders.

However, the hierarchy and directionality between affective and non‐affective cognitive domains remain poorly understood. That is, it is unclear whether deficits in non‐affective cognition contribute to affective cognitive impairment, including emotion dysregulation and impaired face recognition, or whether aberrant affective cognition disrupts non‐affective cognitive performance by taxing executive and attentional resources. Indeed, conscious regulation of emotion relies on higher‐order cognitive abilities such as attention, working memory, and executive function—including inhibitory control and cognitive flexibility—to effectively monitor, interpret, and modulate emotional information and experience [24]. When these functions are impaired, individuals with mood disorders may struggle to manage emotional reactions, leading to heightened emotional reactivity and increased vulnerability to mood episodes. Conversely, intense emotional reactivity can also impair executive performance, partly due to resources prioritizing attention toward highly salient, emotion‐relevant stimuli [25]. Similarly, a longitudinal investigation of children from large‐scale birth cohorts demonstrated that better emotion regulation skills predicted increased executive functioning throughout development but not vice versa [26]. As such, there may be a reciprocal relation between emotion regulation and other emotional cognitive functions on one hand and non‐affective cognition—including executive functions and attention—on the other. However, the hierarchical structure and pattern of inter‐correlations of affective and non‐affective cognitive domains are unclear. This impedes insights into which of the affected domains in mood disorders constitute *core cognitive impairments* that represent the most relevant treatment targets to improve cognition more broadly [27].

Network analysis can clarify how affective and non‐affective cognitive impairments in mood disorders are related. This methodology is well‐suited for modeling many variables simultaneously [28] and often uses conditional dependence, which estimates the relationship between two variables while controlling for all other variables in the network [29]. Despite network analyses of cognitive deficits in mood disorders being scarce, emerging evidence suggests that executive functions may represent a core cognitive impairment [27, 30, 31]. Nevertheless, it is uncertain whether similar patterns will emerge in a network that also includes affective cognitive domains. The study aims to identify (i) the structure of associations between non‐affective and affective cognitive domains across individuals with mood disorders, (ii) the centrality of each domain, and (iii) potential differences in the networks of mood disorders and healthy controls (HC). We hypothesized that executive functions would emerge as a central non‐affective cognitive domain and that this domain would be associated with affective cognitive domains, particularly emotion regulation.

## 2 Materials and Methods

### 2.1 Study Design

The data used for the analysis were pooled from two studies: the Bipolar Illness Onset (BIO) study [32] and the Neurocognition and Emotion in Affective Disorders (NEAD) study [33]. The studies were conducted between 2014 and 2022. Pooling data from these studies was considered appropriate due to the studies' similar inclusion criteria, overlapping cognitive measures, and time points. Both studies have been approved by the Regional Ethics Committee (protocol numbers: H‐7‐2014‐007 and H‐3‐2014‐003). All procedures followed the ethical standards of the national and institutional committees on human experimentation and with the Declaration of Helsinki. All participants provided written informed consent prior to study participation.

### 2.2 Participants

Patients in the BIO study were recruited from the Copenhagen Affective Disorder Clinic, Psychiatric Centre Copenhagen, and patients in the NEAD study were recruited through the Danish Twin Registry, the Danish Psychiatric Central Research Register, and the Danish Civil Registration System. Patients had a diagnosis of MDD or BD type I or II confirmed with the Schedules for Clinical Assessment in Neuropsychiatry (SCAN) interview [34] and were in full or partial remission at the time of inclusion (corresponding to total scores ≤ 14 on the Hamilton Depression Rating Scale 17‐item (HDRS‐17) [35] and the Young Mania Rating Scale (YMRS) [36], respectively). Age and sex‐matched HCs were recruited from the blood bank at Copenhagen University Hospital (BIO) or through the Danish Twin Registry (NEAD) and were included if they had no personal or first‐degree family history of treatment‐required psychiatric disorder. Additional exclusion criteria were pregnancy, a history of brain injury, severe somatic illness, organic mental disorder, current substance use disorder, or having a current mood episode.

### 2.3 Measures

#### 2.3.1 Non‐Affective Cognition

In both studies, the Trail Making Test Part A and B [37] were included as measures of non‐affective cognition, and the Danish Adult Reading Task [38] was included to estimate verbal IQ. In the BIO study, a large neuropsychological test battery was used, including the Letter‐Number‐Sequencing subtest from Wechsler's Adult Intelligence Scale 3rd edition [39], Verbal Fluency with letters S and D [40], Coding and Digit Span Forward from the Repeatable Battery for the Assessment of Neuropsychological Status [41], the Rey Auditory Verbal Learning Test [42], as well as the Spatial Working Memory test and the Rapid Visual Information Processing test from the Cambridge Neuropsychological Test Automated Battery (CANTAB). In the NEAD study, a brief assessment of non‐affective cognition was performed using the Screen of Cognitive Impairment in Psychiatry (SCIP) [43]. The SCIP includes measures of working memory, delayed memory, verbal learning and memory, verbal fluency, and processing speed. The SCIP subtests have been correlated and validated with the neurocognitive tests used in the BIO study [44]. See Table S1 for an overview of the neuropsychological non‐affective cognitive tests.

#### 2.3.2 Affective Cognition

The Facial Expression Recognition Task (FERT) assessed the ability to identify facial expressions [45]. Participants were instructed to press a key corresponding to faces showing six basic emotions (anger, fear, sadness, disgust, happiness, surprise) morphed at 10% intensity levels from a neutral (0%) to a full emotional face (100%). The face stimuli appeared for 500 ms, followed by a black screen. Each emotion at each intensity level was presented four times, resulting in a total of 250 presentations in random order. Accuracy and reaction times were recorded.

The Social Scenarios Task was used to assess emotion reactivity and regulation [46]. The task consists of short written descriptions of highly negative or positive social scenarios and associated self‐belief statements. During the task, participants were instructed to imagine experiencing the social scenarios and either naturally react to or dampen their emotional responses. Participants were asked to rate their levels of pleasantness and unpleasantness/discomfort in the positive and negative scenarios, respectively, on a 100‐point visual analog scale. Each scenario consisted of 11 sentences describing the situation for 3 s, 10 self‐beliefs presented for 3 s, and 10 emotion ratings (pleasantness or unpleasantness).

#### 2.3.3 Functioning

Participants underwent an observer‐rated interview with the Functioning Assessment Short Test (FAST), which assesses six domains of functioning: autonomy, occupational functioning, cognitive functioning, financial issues, leisure time, and interpersonal relationships [47].

### 2.4 Statistical Analysis

For information regarding preprocessing and composite score calculations, see Supporting Information. Clinical and demographic variables were compared across mood disorders and HC using Mann–Whitney U tests for non‐parametric data and independent *t*‐tests for normally distributed data.

#### 2.4.1 Network Estimation

A network was constructed for mood disorders and HC, respectively. The networks were partial correlation networks where each cognitive domain was represented as a *node* and the partial correlations between the items were represented as *edges* [29]. The networks were estimated with the R package *bootnet* [48]. To avoid spurious correlations and reduce the risk of overfitting, the Least Absolute Shrinkage and Selection Operator (LASSO) was used [49]. LASSO shrinks all partial correlation coefficients towards zero and sets smaller weights to zero, resulting in a sparse network. The strength of shrinkage is controlled by the tuning parameter, which is assigned automatically by minimizing the Extended Bayesian Information Criterion (EBIC) [29, 48]. A hyperparameter was manually set to 0.25 for both network analyses, which balances between caution (0) and discovery (0.5).

Finally, to account for the potential effects of subsyndromal depressive symptoms and the differences between illness duration between participants in the two pooled studies (i.e., relatively newly diagnosed vs. established illness), we conducted two additional posthoc sensitivity network analyses using the same approach as above (Figures S5 and S6; Tables S4, S5A,B).

#### 2.4.2 Centrality Indices and Network Density

To assess the importance of each cognitive domain, centrality was measured using strength centrality, which is determined by the sum of weighted connections to other nodes [48]. In addition, the density of each network was calculated, which is the average of edge weights that is, the tendency of cognitive domains to appear simultaneously, reflecting the overall level of connectivity within the network. See Supporting Information for accuracy and stability estimates.

#### 2.4.3 Comparison of Networks

To test for differences between the networks of individuals with mood disorders and HC, the networks were statistically compared. First, Spearman correlations of the adjacency matrices were computed to obtain a coefficient of similarity between the networks. Second, the networks were compared with the R package *NetworkComparisonTest* (NCT) [50]. NCT is a permutation‐based test, and 1000 iterations were run in this test to assess whether the network models differed in global strength (the total sum of signed edge weights) and individual edge strength.

All analyses were performed in R version 4.2.2.

## 3 Results

### 3.1 Sample Characteristics

A total of *n* = 605 participants were included in the analysis (BD, *n* = 302; MDD, *n* = 78; HC, *n* = 225). Demographic and clinical characteristics are presented in Table 1. Individuals with mood disorders and HC did not differ in age or estimated premorbid IQ (*ps* ≥ 0.18). However, individuals with mood disorders had fewer years of education, more subsyndromal depressive and manic symptoms, and presented with greater impairments in functioning relative to HC (*ps* < 0.001). Regarding cognitive variables, individuals with mood disorders displayed lower performance scores in executive function and working memory, attention and processing speed, verbal fluency, as well as less successful emotion regulation and poorer facial expression recognition accuracy (*p*s ≤ 0.05). Groups did not significantly differ in verbal learning or facial expression recognition speed (*p*s ≥ 0.08).

**TABLE 1:** Demographic and clinical characteristics in individuals with mood disorders vs. healthy controls.

	Mood disorders (*n* = 380)	Healthy controls (*n* = 225)	*p*‐value
Demographic variables			
Sex (*n*/%female)	257/67.63%	144/64.00%	0.410
Age years, median (IQR)	31.70 (25.6–39.2)	29.08 (24.4–38.6)	0.182
Education years, median (IQR)	15 (12.1–17)	16 (14–18)	< 0.001***
IQ, mean (SD)	112.38 (5.89)	112.90 (5.68)	0.297
Functioning (FAST), total score, median (IQR)	15 (6–26)	0 (0–2)	< 0.001***
Clinical characteristics			
HDRS, median (IQR)	5 (2–8)	1 (0–2)	< 0.001***
YMRS, median (IQR)	2 (0–4)	0 (0–1)	< 0.001***
Cognition			
Executive function and working memory *z*‐score, mean (SD)	−0.37 (0.85)	0.00 (0.72)	< 0.001***
Attention and processing speed *z*‐score, mean (SD)	−0.35 (0.76)	0.00 (0.69)	< 0.001***
Verbal learning *z*‐score, mean (SD)	−0.13 (0.89)	0.00 (0.86)	0.079
Verbal fluency *z*‐score, mean (SD)	−0.22 (1.09)	0.00 (0.90)	0.007**
Emotion regulation *z*‐score, mean (SD)	−0.15 (0.84)	0.00 (0.89)	0.049*
Facial recognition speed *z*‐score, mean (SD)	−0.06 (1.04)	0.00 (0.84)	0.459
Facial recognition accuracy *z*‐score, mean (SD)	−0.23 (0.94)	0.01 (0.84)	0.001**

*Note:* In bold: *p* values < 0.05. *** < 0.001, ** < 0.01, * < 0.05. Chi‐square for categorical variables, Mann–Whitney for non‐parametric data (median (IQR)), independent *t*‐tests for normally distributed data (mean (SD)).

Abbreviations: FAST, Functioning Assessment Short Test; HDRS, Hamilton Depression Rating Scale 17‐items version; IQ, Intelligence quotient; IQR, interquartile range; YMRS, Young Mania Rating Scale.

### 3.2 Overall Network Characteristics

**FIGURE 1:**
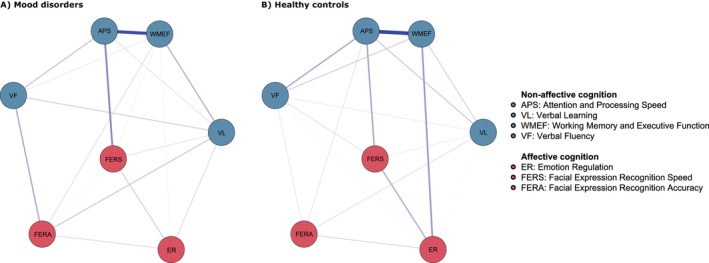
Network models of non‐affective and affective cognitive domains for individuals with mood disorders and healthy controls. Blue nodes represent non‐affective cognitive domains. Red nodes represent affective cognitive domains. Edges between the nodes represent partial correlation coefficients. Thicker edges correspond to stronger associations. Blue edges are positive associations. Red edges are negative associations.

The visualizations of the network models are displayed in Figure 1 and the adjacency matrices are presented in Table S2A,B. The mood disorder and HC networks were both densely connected. The mood disorder network had a density of 0.81 (17/21 edges), while the HC network had a density of 0.86 (18/21 edges). The strongest edge across both networks was between ‘attention and processing speed’ and ‘working memory and executive function’ (mood disorders, *r* = 0.40; HC, *r* = 0.47), being significantly stronger than any other edge in the networks (*p*s < 0.05) (Figure S2). However, confidence intervals of the estimated edge weights were generally wide and overlapping for both networks (Figure S1).

### 3.3 Central Domains

**FIGURE 2:**
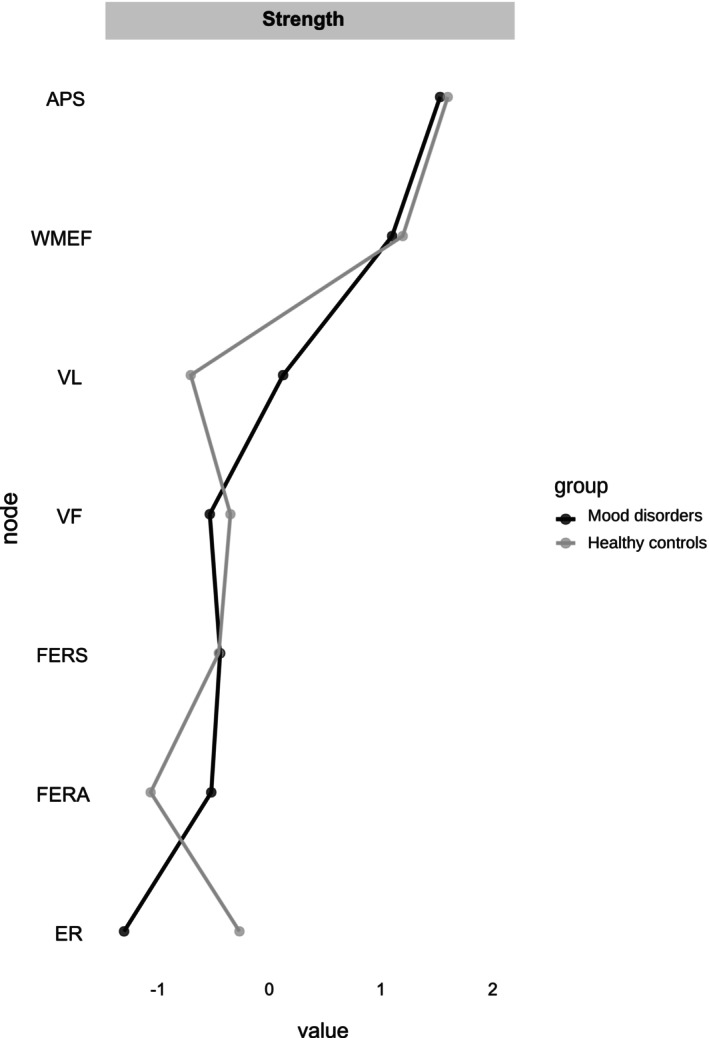
Strength centrality and expected influence of non‐affective and affective cognitive domains for individuals with mood disorders and healthy controls. Centrality measures are in standardized *z‐*scores. APS, attention and processing speed; ER, emotion regulation; FERA, facial expression recognition accuracy; FERS, facial expression recognition speed; VF, verbal fluency; VL, verbal learning; WMEF, working memory and executive function.

In both networks, the domain with the highest strength centrality was ‘attention and processing speed’ (mood disorders, *z* = 1.53; HC, *z* = 1.60), and the second most central domain was ‘working memory and executive function’ (mood disorders, *z* = 1.11; HC, *z* = 1.20) (Figure 2, Table S3). While these two domains were more central than most other domains in the networks, the difference between the domains themselves was not robust in either the mood disorder or the HC network (*p*s > 0.05) (Figure S4). In general, non‐affective cognitive domains were more central than affective cognitive domains. The stability analysis revealed that the strength centrality estimates were stable for both networks (Figure S3). In the mood disorder network, the centrality stability coefficient was 0.60, while in the HC network it was 0.67, which is above the standard cut‐off of 0.50 [48].

### 3.4 Connections Between Non‐Affective and Affective Cognitive Domains

Several associations emerged between affective and non‐affective cognitive domains, with the strongest link observed between ‘attention and processing speed’ and facial recognition speed (mood disorders, *r =* 0.22; HC, *r =* 0.16). A comparable association between working memory and executive function and emotion regulation was observed in the HC network (*r* = 0.19) but was considerably weaker in the mood disorder network (*r* = 0.03). The NCT confirmed that this edge was significantly stronger in the HC network than in the mood disorder network (*p =* 0.02, corrected for multiple testing). Other connections between affective and non‐affective cognitive domains were generally weak (*r*s = 0.03–0.15).

### 3.5 Network Comparison

The weighted adjacency matrices showed a large correlation (*r =* 0.82), indicating that the overall structure of the networks was similar such that strong edges in the mood disorder network tended to also be strong in the HC network. In addition, the NCT showed that the global strength did not differ between the networks (*p* = 0.71), indicating that the overall connectivity was also similar across the two networks. Finally, the comparison of individual edges revealed that only the edge between emotion regulation and ‘working memory and executive function’ was significantly different across the networks (*p =* 0.02). All other edges did not significantly differ between mood disorders and HC (*p*s ≥ 0.50).

## 4 Discussion

The present study used network analysis to explore associations between affective and non‐affective cognitive domains in a large sample of 380 remitted individuals with mood disorders and 225 HC. Partially in line with our hypothesis, the most central cognitive domains in the networks across both groups were ‘working memory and executive function’ and ‘attention and processing speed’. Moreover, we found no support for the hypothesis that executive function is particularly associated with emotion regulation in mood disorders. While the association between ‘working memory and executive function’ and emotion regulation was the strongest edge between non‐affective and affective domains in the HC network, this association was significantly attenuated for individuals with mood disorders. Finally, facial expression recognition speed was associated with attention and processing speed in both mood disorders and HC, with no significant difference between the two groups.

Our findings align with previous research suggesting a central role of executive function and attention in network analyses of symptomatic MDD and BD [30]. The present study extends those findings beyond acute mood episodes. Additionally, studies using traditional mediation and hierarchical regression analyses have shown that impairments in attention, processing speed, and executive functioning account for most of the difficulties in verbal learning and memory observed in mood disorders [51, 52, 53, 54]. Pro‐cognitive interventions targeting one of these domains could, therefore, be expected to benefit the others. Accordingly, improvements in these core domains could have downstream effects on related cognitive functions, including verbal learning and verbal fluency. Hence, these domains should be the focus of pro‐cognitive interventions, especially interventions that target broad cognitive impairments [55]. Moreover, given the interrelation between ‘attention and processing speed’ and facial expression recognition speed across both mood disorders and HC networks, interventions targeting one domain may have a synergistic effect on the other.

Contrary to our findings, previous studies demonstrated an association between executive function and emotion regulation abilities in both mood disorders [56, 57] and healthy populations [58]. While a moderate association between emotion regulation and ‘working memory and executive function’ was found for HC, this relationship was significantly weaker in individuals with mood disorders. A potential mechanism underlying this difference could be the use of different emotion regulation strategies in mood disorders compared with controls. Whereas healthy individuals are likely to use adaptive emotion regulation strategies (e.g., cognitive reappraisal), individuals with mood disorders tend to rely more on maladaptive strategies (e.g., rumination, expressive suppression, and avoidance) and engage less in adaptive strategies ([59]). Cognitive reappraisal (i.e., reframing the emotional meaning of a situation to alter the emotional response) is an effortful strategy that requires working memory and executive function by means of inhibition, updating, and shifting [24]. Accordingly, cognitive reappraisal and working memory rely on overlapping prefrontal neural regions implicated in top‐down cognitive control. Individuals with mood disorders consistently exhibit task‐related hypo‐activity in the dorsal prefrontal cortex during both emotion dysregulation [60, 61, 62] and working memory [63]. In contrast, maladaptive emotion regulation strategies generally lack a conscious and explicit goal and tend to occur automatically without the need for cognitive control [64]. The neural systems involved in maladaptive emotion regulation strategies are not well understood but are thought to rely less on prefrontal cognitive control areas [65]. Participants in the current study were instructed to down‐regulate their emotions using the emotion regulation strategy that they would use in their everyday lives. Thus, the increased reliance on maladaptive strategies that do not engage working memory and executive control could potentially explain the weak association between emotion regulation and ‘working memory and executive function’ in mood disorders. However, as strategy use was not directly assessed in the present study, this interpretation warrants further examination.

Our study has central implications. Firstly, the finding that emotion regulation was only weakly associated with ‘working memory and executive function’ suggests that pro‐cognitive treatments targeting only non‐affective cognition may not directly improve emotion regulation in individuals with mood disorders. Instead, pro‐cognitive treatments should aim to strengthen the link between emotion regulation and working memory and executive function, for example by training effortful and adaptive emotion regulation strategies. Supporting this, one study found that emotion regulation training increased self‐reported use of reappraisal in individuals with BD [66]. While existing pro‐cognitive treatments mainly target non‐affective cognition [67, 68], the present study highlights the relevance of also incorporating affective cognition into pro‐cognitive intervention designs for individuals with mood disorders, who present with impairments in emotion regulation or facial expression recognition [69]. Similar multimodal interventions have previously shown moderate to large effects in individuals with schizophrenia [70], and emerging evidence suggests that individuals with mood disorders may also benefit from affective cognitive training [66, 71]. Notably, the associations identified were independent of mood symptoms, as participants were in remission, and posthoc analyses adjusting for subsyndromal depressive symptoms showed no significant changes in the network (see Supporting Information). However, the pattern of linkages between cognitive domains may differ during depressive episodes, when impairments and maladaptive emotion regulation (e.g., rumination) are more pronounced [72, 73, 74]. Investigating these associations during acute episodes could reveal state‐dependent differences and their contributions to symptom severity.

Strengths of the study include the use of a novel statistical method, assessment of both non‐affective and affective cognition, and a large sample size. Limitations include differences in the patient samples across the pooled studies: patients with BD from the BIO study were recently diagnosed with a shorter illness duration (median = 5 years), whereas those in the NEAD study had more established mood disorders (median = 11.5 years). Nevertheless, sensitivity analyses adjusting for illness duration showed no significant effects (see Supporting Information). Different non‐affective tests were used across studies, and tests of affective cognition did not cover domains like theory of mind, attributional bias, or reward processing, which have previously been shown to be impaired in mood disorders [15, 75, 76, 77]. Lastly, most patients were taking psychotropic medication, which could influence cognition [78].

## 5 Conclusion

This network analysis found that executive function, working memory, attention and processing speed are central domains in mood disorders and that attention and processing speed are specifically associated with facial expression recognition speed. In contrast with HC, ‘working memory and executive function’ was weakly associated with emotion regulation in mood disorders. This highlights the need to strengthen this connection between working memory‐executive function and emotion regulation in future affective cognitive remediation interventions, for example through cognitive control mechanisms that engage executive function in emotionally salient contexts, such as cognitive reappraisal to dampen emotions.

## Author Contributions

Hanne Lie Kjærstad: Conceptualization, methodology, data curation, investigation, writing – original draft, supervision, project administration, writing – review and editing. Astrid Endrup Iversen: formal analysis, investigation, data curation, writing – original draft, writing – review and editing, visualization. Maj Vinberg: investigation, writing – review and editing, supervision, project administration, funding acquisition. Lars Vedel Kessing: investigation, writing – review and editing, supervision, project administration, funding acquisition. Jeff Zarp: writing – original draft, writing – review and editing, supervision. Kamilla Woznica Miskowiak: investigation, methodology, writing – original draft, project administration, supervision, writing – review and editing.

## Funding

The BIO study is funded by grants from the Mental Health Services, Capital Region of Denmark, The Danish Council for Independent Research, Medical Sciences (DFF‐4183‐00570), Weimans Fund, Markedsmodningsfonden (the Market Development Fund 2015–310), Gangstedfonden (A 29594), Helsefonden (16‐B‐0063), Innovation Fund Denmark (the Innovation Fund, Denmark, 5164‐00001B), Copenhagen Center for Health Technology (CACHET), EU H2020 ITN (EU project 722,561), Augustinusfonden (16–0083), Lundbeck Foundation (R215‐2015‐4121). The NEAD study was supported by The Capital Region of Denmark, the Augustinus Foundation, the Axel Thomsen's Foundation, the Lundbeck Foundation (R108‐A10015), the Hoerslev Foundation, and Fonden til Lægevidensskabens Fremme. The sponsors had no role in the planning or conduct of the study or the interpretation of the results.

## Ethics Statement

The authors assert that all procedures contributing to this work comply with the ethical standards of the Committee on Health Research Ethics of the Capital Region of Denmark (protocol no: H‐7‐2014‐007 and H‐3‐2014‐003) and the Danish Data Protection Agency, Capital Region of Copenhagen (protocol no: RHP‐2015‐023) and institutional committees on human experimentation and with the Helsinki Declaration of 1975, as revised in 2008.

## Conflicts of Interest

H.L.K. has received honoraria from Lundbeck. M.V. has received honoraria from Lundbeck, Johnson & Johnson, and Eli Lilly. K.W.M. has received honoraria from Angelini, Lundbeck, and Gideon Richter in the past 3 years. J.Z. has received honoraria from Lundbeck. L.V.K. has, within the recent 3 years, received honoraria from Lundbeck and Teva. The remaining authors have nothing to declare.

## Supporting information


**Data S1:** Supporting Information.

## Data Availability

The datasets analyzed in this study are available from the corresponding author upon reasonable request.
