# Supplementary material for: Exploring the Associations Between Affective and Non‐Affective Cognitive Domains in Mood Disorders and Healthy Controls Using Network Analysis
Source: Acta Psychiatr Scand. 2026 Mar 3;153(5):534–43. doi: 10.1111/acps.70084 (PMC13050598; doi:10.1111/acps.70084)
Supplement: Supplementary file 1 — Data S1: Supporting Information. [file ACPS-153-534-s001.docx]

**Supplementary materials**

**Methods**

**Preprocessing and composite score calculations**

Prior to analysis, all test scores were converted to standardized *z*-scores based on HC data mean and standard deviation (SD). Outlying *z*-scores were truncated such that values >4 SD above or below the mean were transformed to be equal to -4 or +4 (Osborne, 2010). Reaction time scores and scores from Trail Making Test Part A (TMT-A) and B (TMT-B), CANTAB Spatial Working Memory (SWM), and CANTAB Rapid Visual Information Processing (RVP) were inverted such that lower scores reflected poorer test performance.

For non-affective cognition, the *z*-scores of different neurocognitive and Screen for Cognitive Impairment in Psychiatry (SCIP) tests were combined to create four domain-based scores; (i) *Working memory and executive function* (based on WAIS letter-number sequencing, TMT-B, CANTAB SWM (between errors and strategy, respectively) and the Working Memory Test of the SCIP); (ii) *Attention and processing speed* (based on TMT-A, RBANS digit-symbol coding, RBANS digit-span-forward, CANTAB RVP accuracy and mean latency, respectively, and the Processing Speed Test of the SCIP); (iii) *Verbal learning* (based on RAVLT learning (trial I-V correct), immediate recall (trial VI correct), delayed recall, recognition and the immediate and delayed recall scores of the SCIP); and (iv) *Verbal fluency* (based on Verbal Fluency S and D as well as the Verbal Fluency Test of the SCIP).

For affective cognition, three different composite scores were computed. From the Facial Expression Recognition Task (FERT), a score of *discrimination accuracy* was calculated with the formula: ([number of hits + 0.5]/[number of targets + 1]) – ([number of false alarms + 0.5]/[number of distractors + 1]). Reaction times were averaged to obtain a measure of *recognition speed*. Scores from the social scenarios task were arcsine transformed for normality, and a measure of *emotion regulation* was obtained by subtracting the emotional ratings of the dampen trials from the react trials.

After creation of the composite scores, missing data were handled by multiple imputation by chained equations with the R package *mice* (van Buuren & Groothuis-Oudshoorn, 2011), where missing cognitive scores were imputed based on the remaining available cognitive variables. Further, the effect of age and IQ on cognition was controlled for by including these variables in the estimation of the network models and subsequently excluding them from the visualization and analyses, following the procedure from previous network studies (Simpson-Kent et al., 2021). The same approach was applied to control for illness duration (i.e., years) and depressive symptoms (i.e., HDRS-17 total scores) in the supplementary network models.

**Accuracy and stability**

To ensure the robustness of the network results, the accuracy and stability of the estimated network parameters were assessed with the R package *bootnet* (Epskamp et al., 2018). Stability of centrality indices was evaluated by case-dropping subset bootstrap, which repeatedly samples from the data to test the consistency of centrality estimates across iterations. The accuracy of estimated edge weights was also examined using bootstrapped 95% confidence intervals. Additionally, edge-weight difference test was performed on these bootstrapped samples to identify significant differences between any two edges in the networks.

**Network visualization**

The network graphs were visualized using the R package *qgraph* (Epskamp et al., 2012). To enable visual comparison of network structures, a consistent layout was applied across all graphs, and the maximum value was set as the strongest edge in both networks.

| **Table S1**  *Neuropsychological tests within each of the included studies* | | |
| --- | --- | --- |
|  | NEAD | BIO |
| **Working memory and executive function** |  |  |
| WAIS letter-number sequencing |  | x |
| TMT-B | x | x |
| SWM between errors |  | x |
| SWM strategy |  | x |
| SCIP-WMT | x |  |
| **Attention and processing speed** |  |  |
| TMT-A | x | x |
| RBANS digit-symbol coding |  | x |
| RVP accuracy |  | x |
| RVP mean latency |  | x |
| SCIP-PST | x |  |
| **Verbal learning** |  |  |
| RAVLT trial I-V correct |  | x |
| RAVLT trial VI correct |  | x |
| RAVLT delayed recall |  | x |
| RAVLT recognition |  | x |
| SCIP-VLT immediate | x |  |
| SCIP-VLT delayed | x |  |
| **Verbal fluency** |  |  |
| Verbal Fluency S |  | x |
| Verbal Fluency D |  | x |
| SCIP-VFT | x |  |
| **Facial expression recognition** |  |  |
| Facial Expression Recognition Task | x | x |
| **Emotion regulation** |  |  |
| Social Scenarios Task | x | x |
| *Note.* NEAD = Neuromapping of Endophenotypes for Affective Disorders study. BIO = Bipolar Illness Onset study. WAIS = Wechsler Adult Intelligence Scale. TMT = Trail Making Test. SWM = Spatial Working Memory (CANTAB). SCIP = Screening for Cognitive Impairment in Psychiatry. WMT = Working Memory Test. RBANS = Repeatable Battery for the Assessment of Neuropsychological Status. RVP = Rapid Visual Processing (CANTAB). PST = Psychomotor Speed Test. VFT = Verbal Fluency Test. RAVLT = Rey Auditory Verbal Learning Test. VLT = Verbal Learning Test. | | |

| **Table S2A**  *Adjacency matrix mood disorder network* | | | | | | | |
| --- | --- | --- | --- | --- | --- | --- | --- |
|  | APS | VL | WMEF | VF | ER | FERS | FERA |
| APS |  |  |  |  |  |  |  |
| VL | 0.06 |  |  |  |  |  |  |
| WMEF | 0.40 | 0.14 |  |  |  |  |  |
| VF | 0.11 | 0.09 | 0.05 |  |  |  |  |
| ER | 0.00 | 0.07 | 0.03 | 0.00 |  |  |  |
| FERS | 0.22 | 0.06 | 0.03 | 0.00 | 0.09 |  |  |
| FERA | 0.00 | 0.10 | 0.06 | 0.15 | 0.07 | 0.02 |  |
| *Note.* Adjacency matrices were obtained based on the partial correlation matrices of the estimated networks. APS = attention and psychomotor speed, VL = verbal learning, WMEF = working memory and executive function, ER = emotion regulation, FERS = facial expression recognition speed, FERA = facial expression recognition accuracy. | | | | | | | |

| **Table S2B**  *Adjacency matrix healthy control network* | | | | | | | |
| --- | --- | --- | --- | --- | --- | --- | --- |
|  | APS | VL | WMEF | VF | ER | FERS | FERA |
| APS |  |  |  |  |  |  |  |
| VL | 0.10 |  |  |  |  |  |  |
| WMEF | 0.48 | 0.09 |  |  |  |  |  |
| VF | 0.16 | 0.04 | 0.09 |  |  |  |  |
| ER | 0.00 | 0.01 | 0.19 | -0.04 |  |  |  |
| FERS | 0.16 | 0.04 | 0.00 | 0.06 | 0.15 |  |  |
| FERA | 0.05 | 0.06 | 0.00 | 0.05 | 0.08 | 0.00 |  |
|  | | | | | | | |

| **Figure S1**  *Bootstrapped confidence intervals of estimated edge weights* |
| --- |
| **a) Mood disorder network**  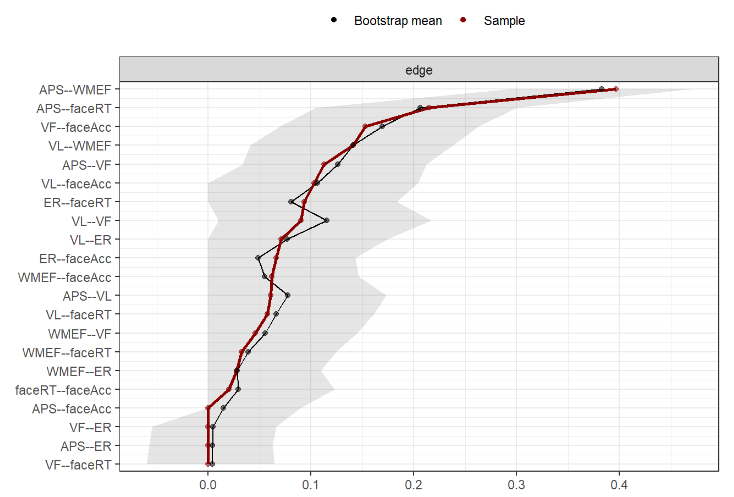  **b) Healthy control network**  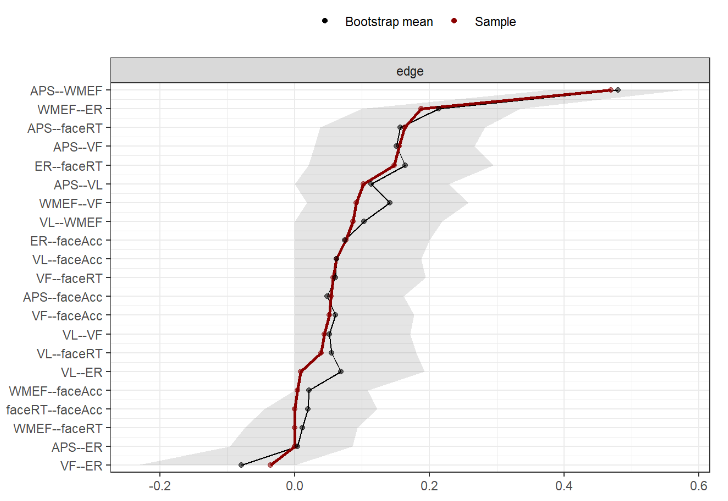 |
| *Note.* Red lines indicate the sample values, black lines show the bootstrapped values, and the grey area represents the 95% bootstrapped confidence intervals (CIs). The horizontal lines correspond to edges, ordered from the highest edge-weight at the top to the lowest edge-weight at the bottom. APS = attention and psychomotor speed, VL = verbal learning, WMEF = working memory and executive function, VF = verbal fluency, ER = emotion regulation, FERS = facial expression recognition speed, FERA = facial expression recognition accuracy. |

| **Figure S2**  *Bootstrapped edge weight difference tests* |
| --- |
| **a) Mood disorder network** |
| 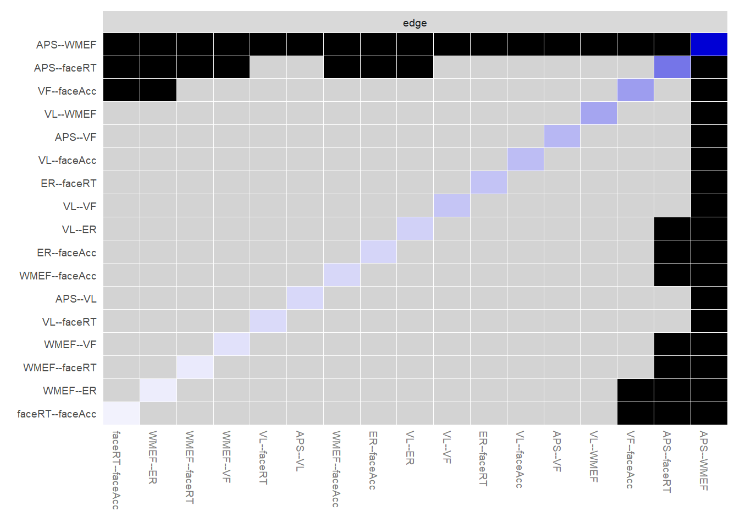  **b) Healthy control network**  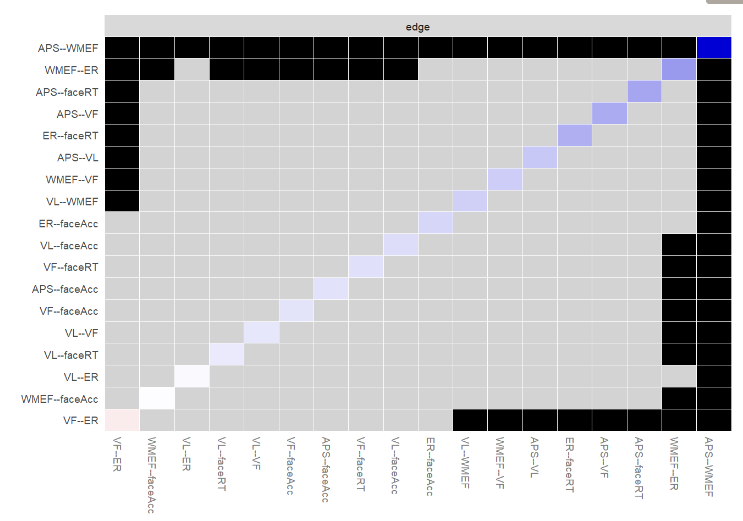 |
| *Note.* Alpha level 0.05. Black squares indicate edges that differ significantly from each other, while grey squares represent edges that do not. APS = attention and psychomotor speed, VL = verbal learning, WMEF = working memory and executive function, VF = verbal fluency, ER = emotion regulation, FERS = facial expression recognition speed, FERA = facial expression recognition accuracy. |

| **Table S3**  *Standardized strength centrality z-scores* | | |
| --- | --- | --- |
|  | Mood disorder  network | Healthy control  network |
| Attention and processing speed | 1.53 | 1.60 |
| Working memory and executive function | 1.11 | 1.20 |
| Verbal learning | 0.13 | -0.70 |
| Verbal fluency | -0.53 | -0.34 |
| Facial expression recognition speed | -0.43 | -0.45 |
| Facial expression recognition accuracy | -0.51 | -1.06 |
| Emotion regulation | -1.29 | -0.26 |
|  |  |  |

| **Figure S3**  *Stability of strength centrality for the mood disorder network (a) and the healthy control network (b).* |
| --- |
| **a) Mood disorder network** |
| 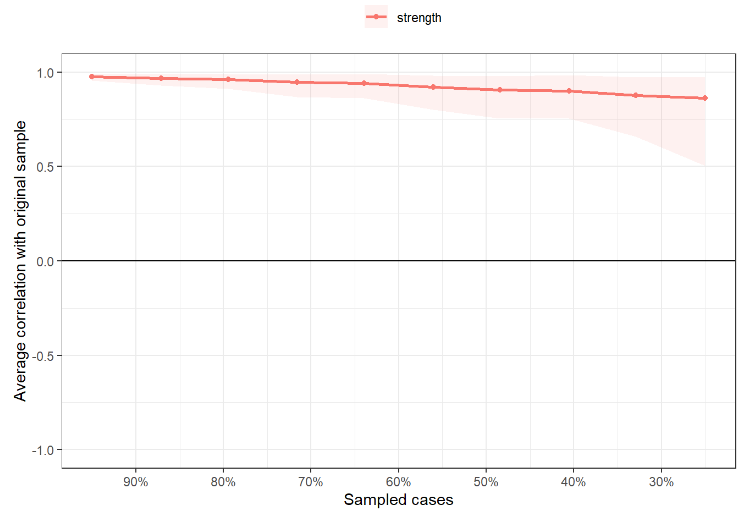  **b) Healthy control network**  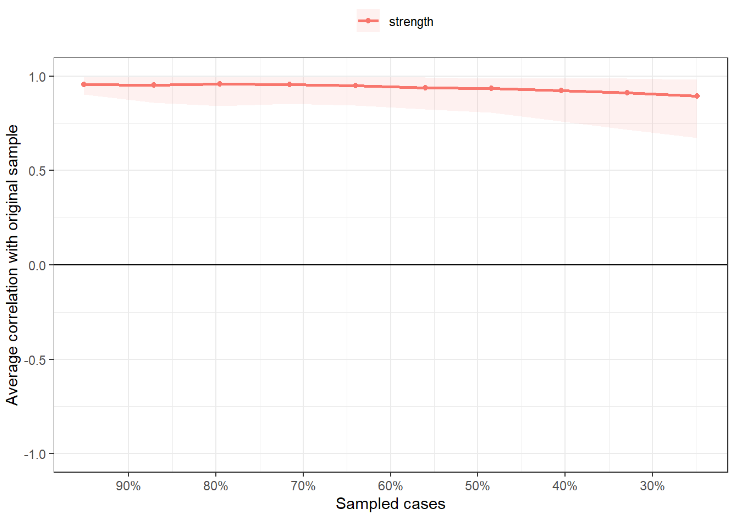 |
| *Note.* Stability is measured as the mean correlation between strength centrality in the original sample and the strength centrality in subsamples with cases removed. The lines show average correlations, while the shaded areas depict the range from the 2.5th to the 97.5th quantile. |
| **Figure S4**  *Centrality difference tests* |
| \| **a) Mood disorder network**  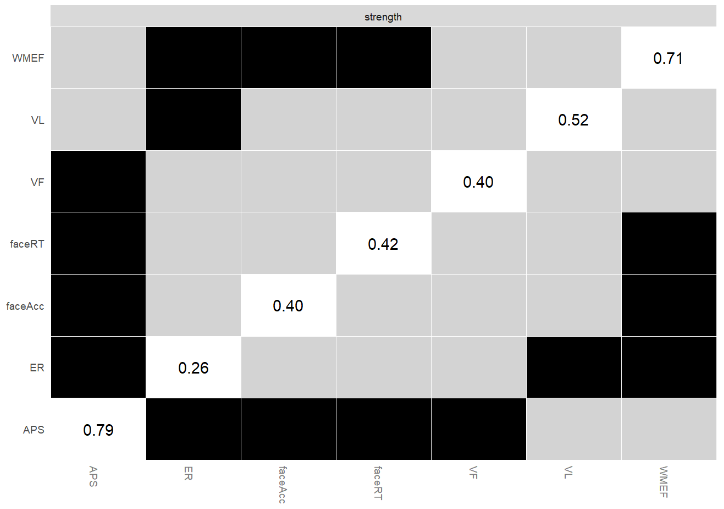  **b) Healthy control network**  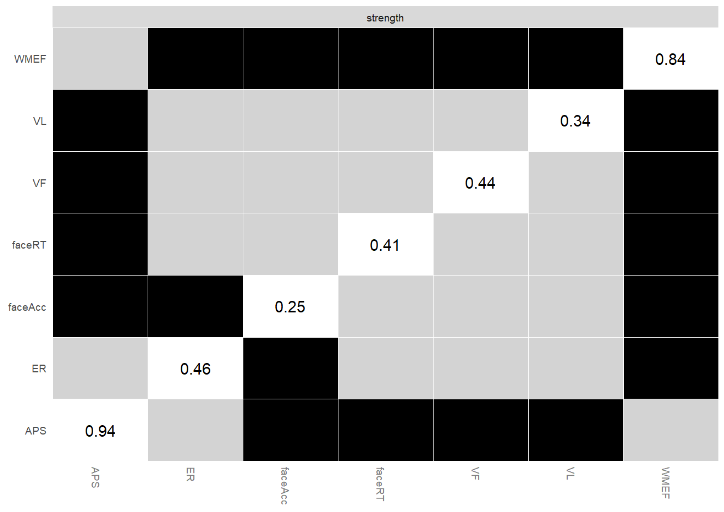 \| \| --- \| \| *Note.* Alpha level 0.05. Black squares indicate nodes that differ significantly from each other, while grey squares represent nodes that do not. White squares display the value of node strength. APS = attention and psychomotor speed, VL = verbal learning, WMEF = working memory and executive function, VF = verbal fluency, ER = emotion regulation, FERS = facial expression recognition speed, FERA = facial expression recognition accuracy. \| |

**Figure S5**

*Network model adjusted for illness duration*

| 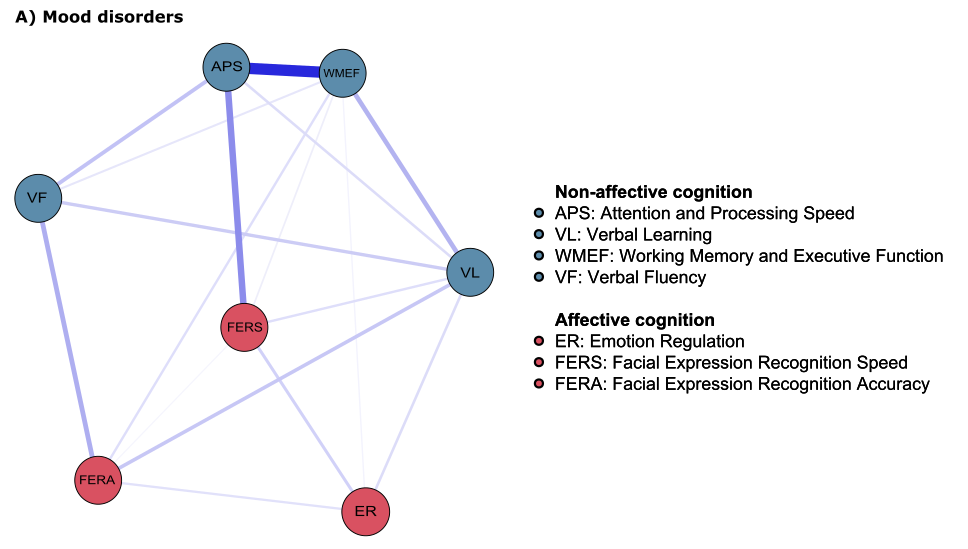 |
| --- |
|  |

**Figure S6**

*Network models adjusted for subsyndromal depressive symptoms*

| 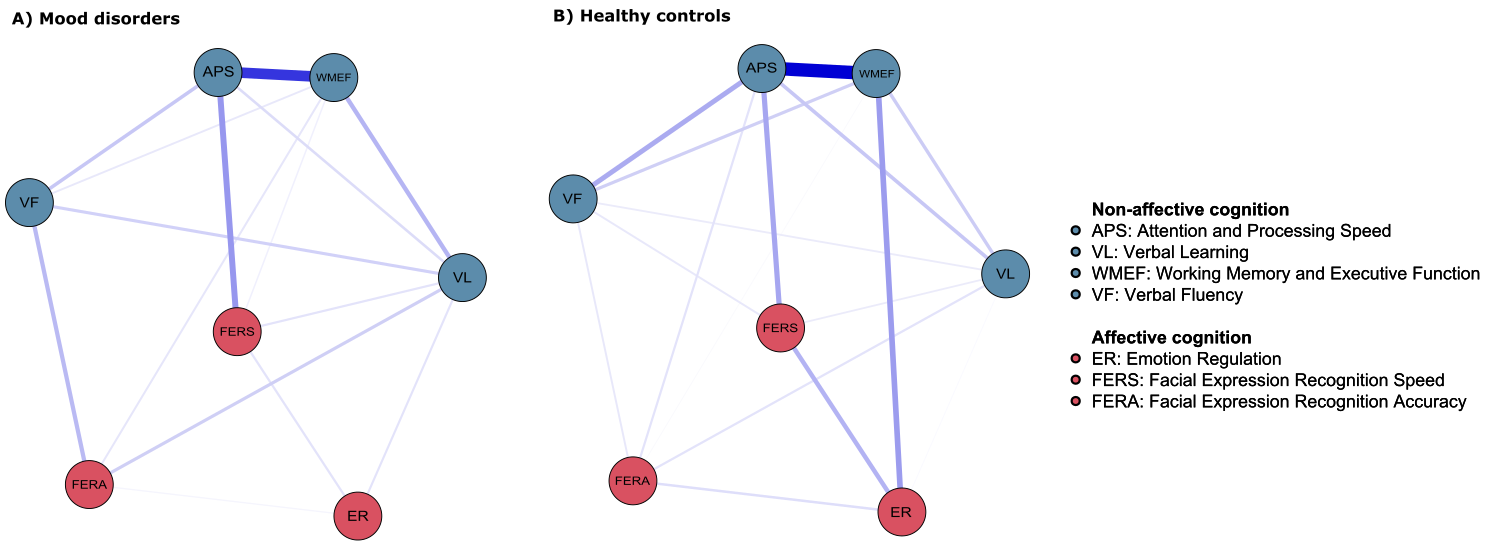 |
| --- |
|  |

| **Table S4**  *Adjacency matrix mood disorder network adjusted for illness duration* | | | | | | | |
| --- | --- | --- | --- | --- | --- | --- | --- |
|  | APS | VL | WMEF | VF | ER | FERS | FERA |
| APS |  |  |  |  |  |  |  |
| VL | 0.06 |  |  |  |  |  |  |
| WMEF | 0.38 | 0.14 |  |  |  |  |  |
| VF | 0.11 | 0.09 | 0.04 |  |  |  |  |
| ER | 0.00 | 0.06 | 0.02 | 0.00 |  |  |  |
| FERS | 0.21 | 0.06 | 0.03 | 0.00 | 0.08 |  |  |
| FERA | 0.00 | 0.10 | 0.06 | 0.14 | 0.05 | 0.01 |  |
|  | | | | | | | |

| **Table S5A**  *Adjacency matrix mood disorder network adjusted for depressive symptoms* | | | | | | | |
| --- | --- | --- | --- | --- | --- | --- | --- |
|  | APS | VL | WMEF | VF | ER | FERS | FERA |
| APS |  |  |  |  |  |  |  |
| VL | 0.06 |  |  |  |  |  |  |
| WMEF | 0.36 | 0.13 |  |  |  |  |  |
| VF | 0.10 | 0.08 | 0.04 |  |  |  |  |
| ER | 0.00 | 0.05 | 0.00 | 0.00 |  |  |  |
| FERS | 0.19 | 0.05 | 0.02 | 0.00 | 0.05 |  |  |
| FERA | 0.00 | 0.09 | 0.04 | 0.12 | 0.02 | 0.00 |  |
| **Table S5B**  *Adjacency matrix healthy control network adjusted for depressive symptoms* | | | | | | | |
|  | APS | VL | WMEF | VF | ER | FERS | FERA |
| APS |  |  |  |  |  |  |  |
| VL | 0.10 |  |  |  |  |  |  |
| WMEF | 0.46 | 0.09 |  |  |  |  |  |
| VF | 0.15 | 0.04 | 0.09 |  |  |  |  |
| ER | 0.00 | 0.00 | 0.18 | 0.00 |  |  |  |
| FERS | 0.16 | 0.03 | 0.00 | 0.04 | 0.13 |  |  |
| FERA | 0.05 | 0.05 | 0.01 | 0.04 | 0.06 | 0.00 |  |
|  | | | | | | | |
